# Rethinking Hip Surgery: A Systematic Review of Sparing Piriformis and Internus, Repairing Externus (SPAIRE) vs. Traditional Hemiarthroplasty Approaches

**DOI:** 10.7759/cureus.89115

**Published:** 2025-07-31

**Authors:** Muhammad Y Raufi, Sagaurav Shrestha, Ward Hamsho, Mahmoud Rhodes

**Affiliations:** 1 Trauma and Orthopedics, Leeds Teaching Hospitals National Health Service (NHS) Trust, Leeds, GBR; 2 Trauma and Orthopedics, Royal Cornwall Hospital National Health Service (NHS) Trust, Cornwall, GBR

**Keywords:** hemiarthroplasty of hip, lateral hardinge approach, neck of femur fractures, orthopaedics trauma, spaire

## Abstract

When elderly patients experience a displaced intracapsular hip fracture, hemiarthroplasty is still the go-to surgical treatment. More recently, a new approach called Sparing Piriformis and Internus, Repairing Externus (SPAIRE) has started gaining attention, in addition to the lateral and anterior approaches already being used. It’s designed to lower the chances of dislocation and help patients recover better after surgery. This review looks at studies that have compared the SPAIRE technique with older, more established surgical methods, especially in terms of things like survival rates, where patients go after discharge, how much pain they feel after surgery, and any complications that come up, like hip dislocations and periprosthetic fractures.

This review was conducted in accordance with the Preferred Reporting Items for Systematic Reviews and Meta-Analyses (PRISMA) guidelines. Eligible studies included randomized controlled trials (RCTs), non-RCTs, and observational studies that compared outcomes between patients undergoing hemiarthroplasty using either the SPAIRE technique or conventional lateral/anterior approaches.

The total number of hips included in our study was 1385. Outcomes assessed included mortality, discharge destination, postoperative mobility, pain scores, and bone mineral density. Comparative evidence suggests that the SPAIRE approach may offer certain short-term advantages over conventional hemiarthroplasty techniques, particularly in terms of early postoperative mobility and pain management. However, these benefits do not appear to translate into significant long-term differences in functional recovery or patient-reported outcomes. Similarly, discharge destinations and bone health indicators show no consistent variation between SPAIRE and other commonly employed surgical approaches. Overall, the available literature indicates that while SPAIRE may provide early postoperative benefits, its long-term outcomes are broadly comparable to alternative methods.

To our knowledge, this is the first systematic review comparing the SPAIRE approach with established surgical techniques in hip hemiarthroplasty. While some outcomes suggest potential advantages with SPAIRE, the current evidence base is limited. Further high-quality, large-scale studies are required to determine its definitive clinical benefit over traditional approaches.

## Introduction and background

Displaced intracapsular fractures of the femoral neck are commonly managed with hip arthroplasty, with hemiarthroplasty being the preferred option in most cases due to patient age and comorbidities [1]. Restoring mobility and functional independence remains a primary concern for patients following hip fracture surgery [2]. In the United Kingdom, the National Institute for Health and Care Excellence (NICE) recommends the lateral approach over the posterior approach for hemiarthroplasty; however, this guidance is underpinned by limited evidence, largely derived from only two studies of low methodological quality [3-5].

The potential for dislocation or nerve injury following hip hemiarthroplasty is of particular concern in frail, elderly patients, as such complications can precipitate rapid functional decline or even contribute to mortality [6]. In light of these considerations, alternative surgical techniques that aim to optimize stability and functional recovery while minimizing soft tissue disruption have gained increasing attention.

One such technique is the Sparing Piriformis and Internus, Repairing Externus (SPAIRE) approach, first described by Hanly et al. [7]. This modification of the traditional posterior (Southern Moore) approach preserves the tendinous insertions of key external rotators, gemellus superior and inferior, obturator internus, and piriformis by developing a dissection plane between gemellus inferior and quadratus femoris. The approach aims to combine the familiarity and extensibility of the posterior method with the functional advantages of muscle preservation.

This systematic review aims to critically evaluate the available literature comparing the SPAIRE technique with conventional approaches, focusing on postoperative outcomes such as return to pre-injury mobility, dislocation rates, and patient-reported outcome measures.

## Review

Methodology

**Figure 1:**
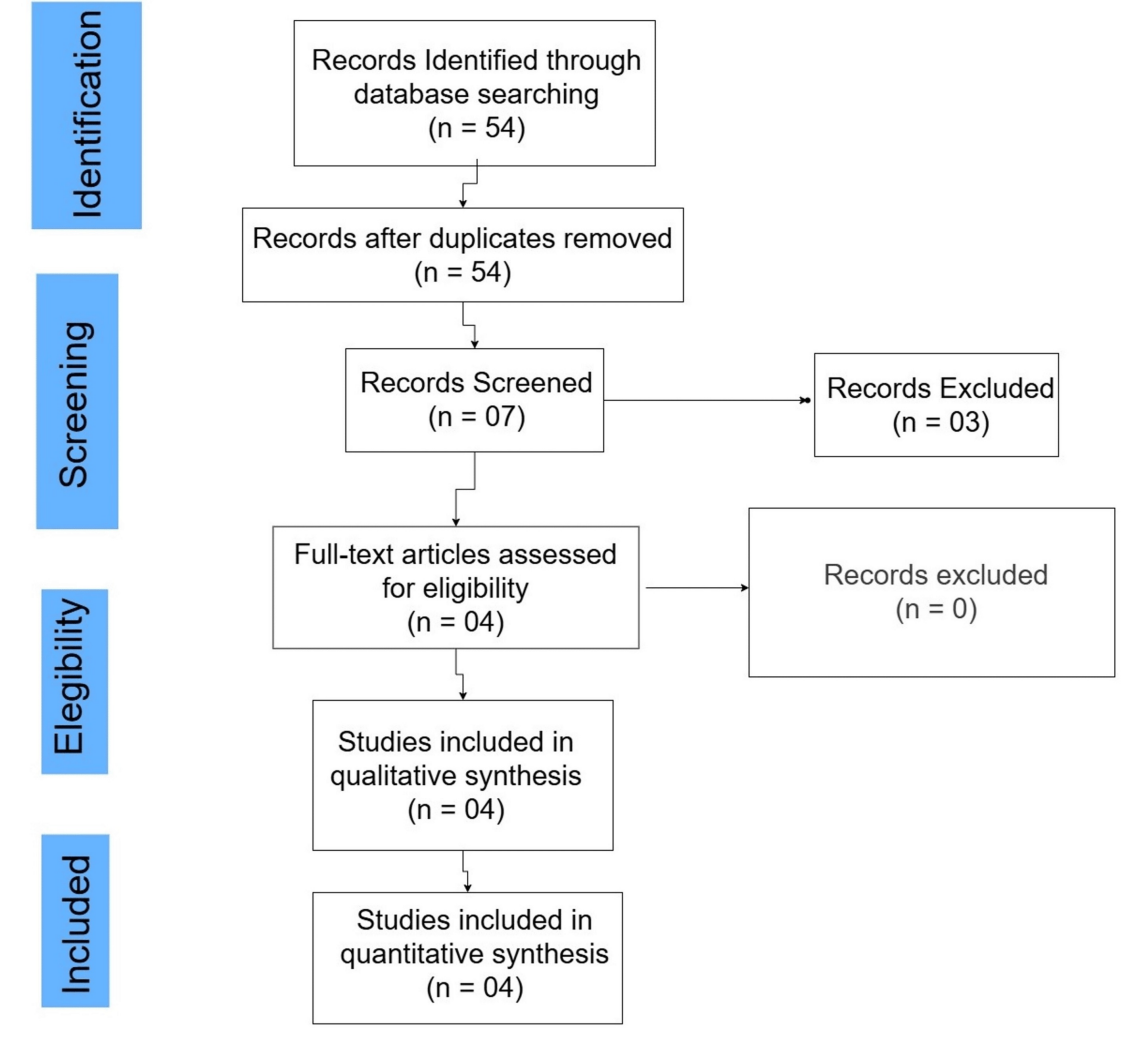
PRISMA flow chart depicting article screening and selection PRISMA: Preferred Reporting Items for Systematic Reviews and Meta-Analyses [8]

This systematic review was conducted according to the Preferred Reporting Items for Systematic Reviews and Meta-Analyses (PRISMA) statement standards (Figure 1) [8].

Eligibility Criteria

We included all comparative studies, randomized controlled trials (RCTs), non-RCTs, and observational studies that evaluated outcomes in patients undergoing the SPAIRE approach for hemiarthroplasty. Eligibility was not restricted by age, sex, or comorbidity status. Studies were excluded if they were systematic reviews or meta-analyses on similar topics or were not published in English.

Literature Search Strategy

A comprehensive, independent literature search was conducted across multiple electronic databases: Medical Literature Analysis and Retrieval System Online (MEDLINE), Embase, Cumulative Index to Nursing and Allied Health Literature (CINAHL), PubMed, Google Scholar, and the Cochrane Central Register of Controlled Trials (CENTRAL). The final search was completed on 15th May 2025. Medical subject headings (MeSH) used included SPAIRE and Hemiarthroplasty. Boolean operators “AND” and “OR” were used to combine search terms appropriately.

Study Selection

An initial search yielded 54 results. A title screening process was performed, from which seven studies were selected for abstract review. Following this, four studies were shortlisted that met all inclusion criteria and were included in the final analysis.

Data Extraction and Management

Data extraction was performed using a customized Microsoft Excel (Microsoft Corporation, Redmond, Washington, United States) spreadsheet, based on the Cochrane data collection form for intervention reviews. A pilot test was conducted on randomly selected articles to refine the extraction tool and ensure consistency and accuracy in data collection.

Assessment of Methodological Quality and Risk of Bias

The methodological quality and risk of bias of all included studies were rigorously assessed. The Newcastle-Ottawa scale [9] was used to assess bias in non-randomized studies across three domains: selection, comparability, and exposure. This scale employs a star scoring system, with a maximum total score of nine stars for each study (Table 1). Additionally, for RCTs, the Cochrane Collaboration’s Risk of Bias Tool [10] was used to evaluate domains such as selection, performance, detection, attrition, reporting, and other biases. Each study was rated as having a low, unclear, or high risk of bias (Table 2).

**Table 1:** Newcastle-Ottawa scale assessing all observational studies for domains of selection, comparability, and outcome Table created by the authors using data from [9]

Study	Selection	Comparability	Outcome	Overall quality	Overall score
Charity et al. [11]	****	**	***	Excellent	9
Davey et al. [12]	****	**	***	Excellent	9

**Table 2:** Risk of bias assessment for randomized controlled trials utilizing the Cochrane Collaboration tool Table created by the authors using data from [10]

First author	Bias	Authors judgment	Support for judgment
Ball et al. [13]	Random sequence generation (selection bias)	Low	Described in detail the random sequence generation method to produce comparable groups
	Selection bias, allocation concealment	Low	Described the method used to conceal the allocation sequence in sufficient detail
	Selective reporting (reporting bias)	Unclear	Insufficient information to permit judgment
	Other bias, other sources of bias	Low	No other bias detected
	Blinding of participants and personnel (performance bias)	Low	Blinding of participants was identified, and also identified that surgeons and theatre staff were not blinded
	Blinding of outcome assessment (detection bias)	Low	Described that a cover sheet was placed on top of the operation note to tell the outcome assessors about the study, and that participants were blinded
	Incomplete outcome data (attrition bias)	Low	Handling of incomplete outcome data was complete and unlikely to have produced bias
Ugland et al. [14]	Random sequence generation (selection bias)	Low risk	Described in detail the random sequence generation method to produce comparable groups
	Selection bias, allocation concealment	Low	Described the method used to conceal the allocation sequence in sufficient detail
	Selective reporting (reporting bias)	Unclear	Insufficient information to permit judgment
	Other bias, other sources of bias	Low	No other bias detected
	Blinding of participants and personnel (performance bias)	Low	Explained how participants were randomized and put into two different groups
	Blinding of outcome assessment (detection bias)	Unclear	Not described in detail
	Incomplete outcome data (attrition bias)	Low	Handling of incomplete outcome data was complete and unlikely to have produced bias

Results

Due to the limited number of shortlisted studies and the heterogeneity of their reported outcomes, it was not feasible to identify a single primary outcome. Consequently, a meta-analysis could not be conducted.

Table 3 summarizes demographic and length of stay (LOS) data. The mean age of patients undergoing the SPAIRE approach was 82.5 years, compared to 83.3 years in the control group. A higher proportion of females was observed in both groups: the SPAIRE group comprised 67.2% females and 32.8% males, while the control group included 64.4% females and 36.7% males.

**Table 3:** Amalgamation table depicting patient demographics SPAIRE: Sparing Piriformis and Internus, Repairing Externus; SD: standard deviation; NR: not reported; RCT: randomized control trial; LOS: length of stay; M: male; F: female

Author and year	Study design	Intervention group number of hips (n)	Intervention group age (mean/years +SD)	Intervention group sex (M:F%)	Intervention treatment (approach)	Intervention group LOS (mean/days + SD)	Control group number of hips (n)	Control group age (mean/years + SD)	Control group sex (M:F%)	Control treatment (approach)	Control group LOS (mean/ days + SD)
Charity et al. [11]	Retrospective cohort	285	85.61 + 7.4	36.5:63.5	SPAIRE	18.2 + 15.06	567	85.44 + 7.7	32.1:67.9	Lateral	19.9 + 16.9
Ball et al. [13]	RCT	122	84.5 +7.4	30.3:69.7	SPAIRE	19.41 + 20.95	122	84.7 + 7.0	32:68	Lateral	19.28 + 14.95
Ugland et al. [14]	RCT	25	80 + 4.7	32:68	SPAIRE	NR	24	82 + 6.0	46:54	Direct anterior	NR
Davey et al. [12]	Retrospective cohort	40	79.9 (No SD mentioned)	32.5:67.5	SPAIRE	11.4 (No SD mentioned)	200	81.2 (No SD mentioned)	32.5:67.5	Lateral	13.5 (No SD mentioned)

LOS for patients managed with the SPAIRE approach ranged from 11.4 to 19.41 days, whereas for the lateral approach, LOS ranged from 13.5 to 19.9 days. No study reported a statistically significant difference in LOS between the two surgical techniques.

Table 4 presents outcomes including mortality, discharge destination, mobility, and pain scores. Three studies reported 120-day mortality [11-13]. Notably, Charity et al. identified a statistically significant difference between the SPAIRE and lateral approaches, with mortality rates of 11.6% and 19.2%, respectively (p = 0.02) [11]. The remaining two studies did not demonstrate a significant difference [12,13].

**Table 4:** Different outcomes, including mortality, discharge destination, mobility, and pain scores NPRS: Numeric Pain Rating Scale; OHS: Oxford Hip Score; BMD: bone mineral density; DA: direct anterior; NR: not reported; SPAIRE: Sparing Piriformis and Internus, Repairing Externus A license to use the OHS was obtained by the authors from Oxford University Innovation Limited.

Study design	30-day mobility to baseline (n (%))	120-day mobility to baseline (n (%))	Discharge destination as pre-fracture residence (n (%))	120-day mortality (n (%))	NPRS at 120 days (Mean + SD)	OHS (Mean + SD)	BMD at 3 months - mean	BMD at 12 months - mean
Charity et al. [11]	NR	NR	SPAIRE = 231 (83.7%), Lateral = 430 (81.1%), P= 0.5	SPAIRE = 33 (11.6%), Lateral = 109 (19.2%), P = 0.02	NR	NR	NR	NR
Ball et al. [13]	NR	NR	SPAIRE = 115 (94.26), Lateral = 116 (95.1), P = 0.24	SPAIRE = 14 (11.5%), Lateral = 19 (15.6%), P = 0.36	SPAIRE = 1.7 + 2.44, Lateral = 2.73 + 3.12, P = 0.03	SPAIRE = 32.24 + 9.27, Lateral = 33.66 + 8.28, P = 0.37	NR	NR
Ugland et al. [14]	NR	NR	NR	NR	NR	NR	SPAIRE = -1.2, DA = -2.3, P = 0.614	SPAIRE = 0.2, DA = -1.2, P = 0.072
Davey et al. [12]	SPAIRE = 37 (91.9), Lateral = 140 (68.4), P = 0.003	SPAIRE = 39 (97.3), Lateral = 175 (86.8), P = 0.09	SPAIRE = 39 (97.5%), Lateral = 188 (94%), P = 0.70	SPAIRE = 4 (10%), Lateral = 31 (15.5%), P = 0.47	NR	NR	NR	NR

Three studies [11-13] also assessed discharge destination relative to pre-fracture residence; none found a statistically significant difference between the SPAIRE and lateral approaches.

Davey et al. reported on return to baseline mobility at 30 and 120 days postoperatively. Their findings showed a statistically significant improvement in 30-day mobility with the SPAIRE approach (p = 0.003). However, no significant difference was observed at 120 days (p = 0.09) [12].

Ball et al. evaluated postoperative pain and functional outcomes using the Numeric Pain Rating Scale (NPRS) and Oxford Hip Score (OHS). Their study demonstrated a statistically significant reduction in postoperative NPRS in favor of the SPAIRE approach (p = 0.03), while no significant difference was found in OHS (p = 0.37) [13].

Ugland et al. compared the SPAIRE and direct anterior approaches with regard to bone mineral density (BMD) at three and 12 months postoperatively. They found that the differences in BMD were not statistically significant at either time point. Both the SPAIRE and direct anterior groups showed an increase in mean BMD from -1.2 to 0.2 and -2.3 to -1.2, respectively [14].

In terms of complications, Charity et al. reported two periprosthetic fractures in the lateral group and one in the SPAIRE group. The lateral group cases required open reduction and internal fixation (ORIF), while the SPAIRE case underwent revision surgery. Additionally, three periprosthetic infections were noted, two in the lateral group and one in the SPAIRE group. Hip dislocation occurred in three patients from the lateral group and one from the SPAIRE group [11].

Davey et al. reported two dislocations and one revision surgery in the lateral group, with no such events occurring in the SPAIRE group [12].

Discussion

The SPAIRE approach was first introduced by Hanly et al. in 2017 [7]. Since its introduction, the technique has gained popularity among orthopedic surgeons due to its muscle-sparing nature, specifically preserving the piriformis and obturator internus muscles while allowing for repair of the obturator externus.

The functional significance of the short external rotators of the hip, namely the piriformis, gemellus superior, obturator internus, and gemellus inferior, was demonstrated by Vaarbakken et al. through a cadaveric study. Their findings suggested that these muscles serve as primary extensors and abductors of the flexed hip, functions that are essential during activities such as rising from a seated position or ascending stairs [15].

Charity et al. highlighted that preserving the short external rotators along with the gluteus medius and minimus using the SPAIRE approach may enhance postoperative recovery in patients undergoing hip hemiarthroplasty [11]. In contrast, the conventional direct lateral approach often involves splitting or detachment of a substantial portion of the gluteus medius and minimus, which may adversely affect postoperative hip function.

Furthermore, Charity et al. proposed that combining the SPAIRE approach with robust capsular repair could provide sufficient joint stability, enabling patients to mobilize fully and bear weight without restriction [11].

Both Charity et al. and Davey et al. reported a lower incidence of postoperative dislocation with the SPAIRE technique [11,12], suggesting a potential advantage in reducing dislocation risk and improving functional outcomes in this often frail patient cohort [11].

Ugland et al. investigated whether the surgical approach, SPAIRE versus direct anterior, impacts periprosthetic BMD [14]. Their analysis was based on an RCT by Merle et al., which previously reported greater bone loss with the direct lateral approach compared to the anterolateral [16]. However, Ugland et al. found no statistically significant difference in BMD between the SPAIRE and direct anterior approaches [14].

Ball et al. conducted a comparative study evaluating the SPAIRE versus the standard lateral approach in patients undergoing hemiarthroplasty for displaced intracapsular femoral neck fractures. Using the OHS at 120 days postoperative as the primary outcome measure, they found no statistically significant difference between the two groups. Secondary outcomes, including mobility, function, and quality of life, were also comparable in both the short and long term. Notably, patients treated with the SPAIRE approach reported less postoperative pain in the early recovery phase, as measured by the NPRS [13].

Charity et al. and Davey et al. examined postoperative complications associated with the SPAIRE and lateral approaches, focusing on periprosthetic fractures, infections, and dislocations. Although the overall number of periprosthetic fractures was low, the single case in the SPAIRE group required revision surgery, potentially indicating a more severe injury despite its lower frequency compared to the lateral group [11,12].

In terms of periprosthetic infections, slightly higher rates were observed in the lateral approach; however, the limited number of cases restricts the ability to draw definitive conclusions [11]. Hip dislocations were consistently more common in the lateral group across both studies, lending support to the hypothesis that the SPAIRE technique may confer improved postoperative joint stability [11,13].

Overall, the findings suggest that the SPAIRE approach may be associated with a lower incidence of dislocations and infections and a comparable or reduced rate of serious complications such as fractures and the need for revision surgery. Nevertheless, the small sample sizes and low event rates underscore the necessity for larger, high-quality studies to validate these preliminary observations [11,13].

In conclusion, while the SPAIRE approach remains a relatively novel technique in hip hemiarthroplasty, preliminary evidence suggests that it is at least non-inferior to the standard lateral and direct anterior approaches in terms of clinical outcomes. However, further high-quality, large-scale studies are needed to determine its definitive role and potential superiority in specific patient populations.

Limitations

The SPAIRE technique is relatively novel, and consequently, there is a limited body of research available on its outcomes. As a result, insufficient data exists to definitively determine whether the SPAIRE approach is superior to the lateral approach. Additionally, the literature search was restricted to articles published in English, which may have excluded relevant studies available in other languages.

## Conclusions

To our knowledge, this is the first systematic review comparing the SPAIRE approach with other surgical techniques in hip hemiarthroplasty. Current evidence remains limited and does not support a definitive conclusion regarding the superiority of the SPAIRE approach over traditional methods. Due to this reason and high heterogeneity, a meta-analysis was not possible. As a relatively recent innovation, the SPAIRE technique warrants further high-quality research. Additional prospective studies and RCTs are necessary to enable more comprehensive systematic reviews and meta-analyses in the future.
